# Natural Terpenes as Penetration Enhancers for Transdermal Drug Delivery

**DOI:** 10.3390/molecules21121709

**Published:** 2016-12-11

**Authors:** Jun Chen, Qiu-Dong Jiang, Ya-Ping Chai, Hui Zhang, Pei Peng, Xi-Xiong Yang

**Affiliations:** 1 Hubei Collaborative Innovation Center of Targeted Antitumor Drug, Jingchu University of Technology, Jingmen 448000, China; pengpei2011@126.com; 2 Pharmaceutical Research Laboratory of Chinese Medicine, School of Pharmacy, Nanjing University of Chinese Medicine, Nanjing 210023, China; qiudong_J@126.com (Q.-D.J.); 15951837195@163.com (Y.-P.C.); zh20150908@sina.com (H.Z.)

**Keywords:** terpenes, penetration enhancer, transdermal drug delivery, stratum corneum

## Abstract

The greatest hindrance for transdermal drug delivery (TDD) is the barrier property of skin, especially the stratum corneum (SC). Various methodologies have been investigated and developed to enhance the penetration of drugs through the skin. Among them, the most popular approach is the application of penetration enhancers (PEs), including natural terpenes, a very safe and effective class of PEs. In the present paper, we focused on terpenes as skin PEs for TDD. The mechanism of their action, the factors affecting their penetration enhancement effect, as well as their possible skin toxicity were discussed. Terpenes abundant in nature have great potential in the development of PEs. Compared to synthetic PEs, natural terpenes have been proved to possess higher enhancement activity. Interaction with SC intercellular lipids is the main mechanism of action for terpenes. The key factor affecting the enhancement effect is the lipophilicity of both terpenes and drug molecules. In addition, a lot of terpenes have also been proved to be much less toxic compared to azone, the classic synthetic PE. In summary, terpenes may be preferred over the chemically synthesized compounds as safe and effective PEs to promote the percutaneous absorption of drugs.

## 1. Introduction

Transdermal drug delivery (TDD) has become a viable alternative to conventional routes of drug administration since it can avoid the hepatic first pass effect, improve the compliance of patients, decrease the administration frequency, and reduce the gastrointestinal side effects. Despite its great potential, delivery of most drug molecules via a transdermal route remains one of the major challenges in the development of transdermal drug delivery systems (TDDS). The principal barrier to TDD is located in the stratum corneum (SC), the outermost layer of the skin, thereby limiting percutaneous absorption [1]. The SC is composed of 15~20 layers of flattened cells with no nuclei and cell organelles separated by an intercellular lipid domain. The structure of the SC can be described in terms of a so-called “brick-and-mortar” model, with the keratin-filled corneocytes as the bricks and the intercellular lipids as the mortar. The lipids, comprised of 50% ceramides, 25% cholesterol, 15% free fatty acids, as well as low levels of phospholipids [2], are organized in orderly-arranged lamellar layers and thus form an impermeable barrier to drug diffusion [2,3,4,5].

There are mainly three possible routes for percutaneous penetration of drug molecules, which include intracellular diffusion across the SC corneocytes, permeation through the SC intercellular lipid spaces, and penetration through skin appendages [6]. Among these options, the scientific community agrees that the intercellular lipid domain of the SC is the main pathway for the skin penetration of most drug molecules [1,7].

To achieve therapeutically effective drug levels at the proper site following TDD, the barrier properties of the SC must be modified to enable sufficient drug permeation. A lot of approaches have been used to alter the SC barrier properties, and the most commonly applied approach is the application of penetration enhancers (PEs), which have been used in TDDS since the 1960s [8]. Until now, efforts have been directed at identifying desirable PEs which possess safe yet effective properties.

Due to their high enhancement effect and low skin irritation, terpenes of natural origin are now receiving much attention in pharmaceutical and cosmetic formulations as PEs [8,9]. Terpenes, primarily extracted from medicinal plants, are volatile compounds with molecular components that are composed of only carbon, hydrogen and oxygen atoms. The basic chemical structure of terpenes consists of a number of repeated isoprene (C_5_H_8_) units which are used to classify terpenes. They are generally regarded to be safer compared to synthetic Pes which include surfactants, fatty acids/esters, and solvents [9]. Furthermore, a few terpenes (e.g., 1,8-cineole, menthol, and menthone) are included in the list of Generally Recognized As Safe (GRAS) agents issued by the US Food and Drug Administration [10].

This review aims to give an overview of terpenes as PEs for use in TDD, which will be helpful to researchers working on TDDS in the selection of a suitable terpene.

## 2. Skin Penetration Enhancement Effect

Many publications have already provided substantial evidence that terpenes are capable of enhancing percutaneous absorption [11,12,13,14,15,16].

Compared to conventional synthetic PEs (e.g., oleica acid, azone, dimethyl sulfoxide (DMSO), ethanol), natural terpenes have been shown to improve the permeation of both lipophilic and hydrophilic compounds. One study was focused on bulfalin which is a drug molecule suitable for TDD (molecular weight = 386.5, log P = 2.78). The feasibility of using different PEs to reduce the permeation barrier was evaluated. The results of skin permeation studies of bulfalin demonstrated that terpenes (1,8-cnieole, d-limonene, and l-menthol) were the most effective among different PEs. The enhancement ratios (ERs) of 5% 1,8-cineole, d-limonene, and l-menthol were determined to be 17.1, 22.2, and 15.3, respectively. In comparison, other synthetic PEs at the same concentration increased the flux of bulfalin by less than 6-fold. The ER values were determined to be 5.1, 5.2, 2.5, 2.4, 1.4, and 5.3 for oleic acid, lauric acid, SDS, azone, ethyl oleate, and ethyl lauric acid, respectively [11]. In another study, different PEs were incorporated into the gel to improve the skin permeation of hydrophilic lidocaine hydrochloride. The ER values of DMSO, urea, sodium lauryl sulfate (SLS), and menthol were determined to be 1.13, 1.72, 2.59, and 3.72, respectively [12]. In addition, the synergistic effect of terpenes and iontophoresis has been demonstrated and utilized to increase the percutaneous absorption of oligonucleotides [13].

The effects of PEs on the bioavailability of meloxicam (MLX) gels were investigated and compared after TDD to rabbits. After application of the control gel without PE, the drug was detectable but not quantifiable in plasma. In contrast, after administration of the gel containing 5% menthol, MLX appeared in plasma immediately and reached the maximum peak concentration in about 4 h. It was found that the 5% menthol gel delivered 3.93 ± 0.85 mg of MLX into the systemic circulation compared to 1.41 ± 0.24 mg of MLX delivered by 1% oleic acid gel [14].

Using 1% 1,8-cineole as a PE, valsartan transdermal gel was prepared and evaluated. The pre-clinical evaluation of the antihypertensive efficacy of the valsartan gel was carried out using experimental hypertensive rats. The gel was applied to the rat abdominal skin area and the blood pressure values from the tail were recorded at different intervals up to 24 h. The valsartan gel containing 1% 1,8-cineole was found to reduce the blood pressure remarkably (*p* < 0.001) to about the normal value and maintain its level for 24 h. Conversely, no reduction in blood pressure was observed with the control gel without a PE [15].

Propranolol hydrochloride (PH) can be used to treat infantile hemangiomas. To formulate the PH gel, nine terpenes were compared and 3% farnesol was found to be the most effective. The final PH gel used hydroxypropyl methylcellulose (HPMC) as the matrix material and used 3% farnesol as the PE. In clinical tests, the PH gel was proved to be an effective treatment option for superficial infantile hemangioma considering its wonderful clinic efficacy without obvious side effects [16].

The terpenes used to increase drug penetration are summarized in Table 1. As PEs, the most commonly used terpenes include 1,8-cineole, menthol, limonene, menthone, nerolidol, and others. It should be noted that 25 out of 28 (89.29%) terpenes are oxygen-containing terpenes.

The penetration enhancement effect of these terpenes is summarized in Table 2.

It should be emphasized that the penetration enhancement effect of terpenes on the SC may be different in different vehicle systems due to the differences in physico-chemical properties of these solvents and their interactions with the SC [16]. Co-solvents like propylene glycol (PG) or ethanol have synergistic effects when added to the terpenes. In addition, other factors including skin type, pH values, and formulation ingredients should also be taken into account as the sources of experimental variabilities.

## 3. Mechanism of Action

It is widely agreed that PEs may enhance the skin penetration of a drug molecule by acting on the SC intercellular lipids via extraction or fluidization and/or by increasing the SC partitioning of the drug and/or by modifying the keratinized protein conformations [10]. As demonstrated in Table 2, terpenes possess high enhancement activity for both hydrophilic and lipophilic drugs even at low concentration. It seemed that terpenes interact with SC components by more than one mechanism, although their interactions with SC intercellular lipids could be the key mechanism.

### 3.1. Effect on SC Lipids

The effect of terpenes on SC lipids mainly involves the interactions at two sites, namely the lipophilic tails of the intercellular lipid and the polar head groups, affecting both lipoidal intercellular and polar transcellular pathways. Nowadays, the former route has attracted more attention.

To elucidate the mechanism of action, attenuated total reflection-fourier transform infrared spectroscopy (ATR-FTIR) or FT-IR spectrometry studies were often applied to investigate the biophysical alterations of the skin barrier which could be attributed to its capability of obtaining the conformation information of the SC lipids and keratins. Stretching peaks near 2850 cm^−1^ (C-H symmetric stretching absorbance frequency peak), 2920 cm^−1^ (C-H asymmetric stretching absorbance frequency peak), 1640 cm^−1^ (Amide I), and 1540 cm^−1^ (Amide II) are usually detected following the administration of terpenes to the SC [33]. The shift to a higher frequency of C-H stretching peaks (~2850 and ~2920 cm^−1^) occurs when methylene groups of the SC lipid alkyl chains change from *trans* to *gauche* conformation, indicating the perturbation of SC lipids. The stronger the perturbation, the higher the C-H stretching peak position. The areas and heights of these two peaks (~2850 and ~2920 cm^−1^) are proportional to the amount of the SC lipids. So any extraction of the lipids by terpenes results in a decrease of peak area and peak height.

Both menthol and menthone, the classic terpenes as PEs, can enhance the skin permeability by extracting SC lipids [31,32]. Revealed by ATR-FTIR studies, compared with the control, the shift of asymmetric or symmetric C-H stretching to higher wave number was observed after treatment with menthol or menthone, despite the fact that the alteration of these peak positions was relatively weak. The results indicated that menthol and menthone could slightly interact with the lipophilic tails of skin lipids, which could contribute to the transdermal absorption of lipophilic drugs. Remarkably, menthol and menthone resulted in the significant decrease of peak areas of C-H stretching absorption peaks, indicating that they could directly extract part of the SC lipids to weaken the skin permeability barrier provided by the SC lipids. Moreover, no significant difference in the peak positions nor peak areas of two amide bonds could be observed after treatment with menthol and menthone, suggesting they had little effect on the keratin in corneocytes [32]. In addition, it was demonstrated that the capacity of menthone in disturbing and extracting lipids was higher than that of menthol and azone [31].

Similar results were obtained with other terpenes [28,29]. There were obvious differences in the FT-IR spectra of the control and the terpene-treated (1,8-cineole, 1,4-cineole, rose oxide, safranal, and valencene) SC samples. Considering the peak height and area of asymmetric and symmetric C-H stretching peaks, these terpenes were demonstrated to enhance permeation of valsartan by directly extracting SC lipids [28]. However, they did not fluidize the SC lipids as the peak shift to a higher wave number was not observed [29]. 

For other terpenes, the mechanism might be reversed. ATR-FTIR study results showed nerolidol produced significant blue shift of asymmetric and symmetric C-H stretching peaks. But the decrease of peak heights and areas for CH_2_ asymmetric and symmetric stretching peaks were statistically insignificant. It could be concluded that nerolidol fluidized rather than extracted the SC lipids [30]. In order to further investigate the interaction between SC lipids and terpenes, molecular dynamics simulations could be used to reveal the detailed mechanism [34].

### 3.2. Effect on Hydrogen Bond Connection

A large number of ceramides are tightly arranged in the SC lipid bilayer via hydrogen bonding. It is the hydrogen bond connection that forms the network at the head of ceramide. The hydrogen bonding makes the lipid bilayer strong and stable, and it is needed in order to maintain the barrier trait of the SC. The tight network may be loosened by the terpenes with a functional group that can donate or accept a hydrogen bond.

It was found that menthol had the better penetration enhancement effect on ligustrazine hydrochloride than that of menthone. The structures of menthol and menthone only differed by the attached group. The hydroxy group of menthol forms a hydrogen bond with an amide group, which is much easier to form than with the ketone group of menthone. This loosens the network of SC to improve the permeation flux of the drug [31].

ATR-FTIR studies using a simple SC lipid model have revealed that the presence of 1,8-cineole and L-menthol reduces the amide I stretching frequency, indicating that they act mainly on polar lipid headgroups and break inter- and intra-lammellar hydrogen bonding networks [10,35].

Among the terpenes evaluated (menthol, nerol, camphor, methyl salicylate), nerol was found to produce the highest level of disruption of the SC lamellae. A hydroxyl group in the terpene molecule can form a hydrogen bond, leading to disruption of existing hydrogen bonds between the ceramide head groups in the SC bilayer [5].

### 3.3. Effect on SC Partition of Drugs

The partition of drug molecules into the SC is the first step of transdermal drug delivery, and it lays down the foundation for penetration enhancement. Therefore, increasing the partition coefficient has become one of the action mechanisms of PEs [36]. A positive correlation between terpene uptake (menthol, thymol, carvacrol, menthone and cineole) into the SC intercellular lipid and β-estradiol partitioning enhancement was found. This indicated that terpene dissolved in the intercellular lipid domain can help to improve drug partitioning into the SC [22].

To measure the SC partition, the dried SC sheets were pulverized into powders. The partition coefficient of propranolol hydrochloride in the SC powder with terpenes ((+)-borneol, (+)-camphor and α-bisabolol) was found to be significantly higher than that with vehicle (*p* < 0.05). It is suggested that the interaction between the drug and terpenes via hydrogen bonding contributes to the enhancement of the partition coefficient. Following treatment with terpenes, the concentration of PH in the SC increased as a result of a molecular complex formation between the drug and the terpene [19]. 

Modelling studies suggest that either hydrocarbon or oxygen-containing terpenes could form complexes with drugs. It was proposed that hydrocarbon terpenes could interact with drug molecules by donor/acceptor interactions, van der Waals forces, and HBD (hydrogen bond donor)-π interactions, while oxygen-containing terpenes could interact with drugs by forming hydrogen bonds [37].

The effect on the SC partition may depend on the lipophilicity of the drug. A series of model drugs with a wide span of lipophilicity, namely indometacin (log P = 3.80), lidocaine (log P = 2.56), aspirin (log P = 1.23), antipyrine (log P = 0.23), tegafur (log P = −0.48), and 5-fluorouracil (log P = −0.95) were employed to study the penetration enhancement effects of camphor. The enhancement ratios of the SC/vehicle partition coefficients of model drugs were measured to be 1.68 (indometacin), 2.04 (lidocaine), 1.21 (aspirin), 0.98 (antipyrine), 1.05 (tegafur), and 0.96 (5-fluorouracil). It was indicated that lipophilic camphor could facilitate the partition of lipophilic drugs into the SC [21].

### 3.4. Effect on Physiological Reactions

Indeed, some terpenes can induce physiological reactions in the living skin, such as vasodilatation and increase of skin temperature, that can affect their efficacy as PEs. 

Menthol’s ability to chemically trigger the cold-sensitive transient receptor potential cation channel subfamily M member 8 (TRPM8) receptors in the skin is responsible for the well-known cooling sensation after its application to the skin. In addition, menthol can stimulate skin nociceptors and initiate an axon reflex with subsequent release of vasodilator peptides [38]. Moreover, an increase in skin temperature has been found after dermal administration of a mixture containing menthol [39].

## 4. Factors Affecting the Penetration Enhancement Effect

### 4.1. Lipophilicity of the Drug

The skin permeability of drug molecules is closely associated with their physicochemical properties, such as lipophilicity, molecular weight, and melting point. It is generally accepted that the optimal logP for a drug to penetrate the SC is in the range of 1~3 and the upper limit on MW is about 500 [40]. A quantitative structure-activity relationship (QSAR) model had been proposed to predict skin permeability of the drug: log *k_p_* = −6.3 + 0.71 log P − 0.061 MW (*r*^2^ = 0.67), where *k_p_* is the skin permeability coefficient, and MW is the molecular weight [41]. Based on the model, the drug lipophilicity appears to be the predominant factor affecting the skin permeability of drugs.

A parabolic curve relationship was found to exist between logP values of model drugs and the ER values of terpenes. The ER of limonene was in a parabolic curve relationship roughly with the lipophilicity of model drugs (ER = −4.89 (log P)^2^ + 12.34 log P + 25.87, *r* = 0.682), implying that limonene could achieve the optimum permeation effect for moderate lipophilic drugs (an estimated log P value of 1.0) [25]. Similar results were obtained with borneol [20]. The correlation analysis displayed that the ER values were roughly in a parabolic curve relationship with the logP values of model drugs, 1% borneol: ER= −0.46 (log P)^2^ + 0.41 log P + 5.18 (*r* = 0.86); 3% borneol: ER = −1.57 (log P)^2^ + 2.64 log P + 13.58 (*r* = 0.79); 5% borneol: ER= −2.46 (log P)^2^ + 4.43 log P + 21.37 (*r* = 0.70). Based on the analysis results, borneol could achieve the optimum permeation-enhancing performance for moderately hydrophilic drugs (an estimated logP value of −0.5~0.5). A parabolic curve was also obtained after plotting the ER of camphor against the drug log P values [21]. The optimal regression equation was obtained from regression analysis: ER= −0.43 (log P)^2^ − 1.26 log P + 13.95 (*r* = 0.86), indicating that the best log P value of the drug was about 0 to obtain the highest penetration enhancement effect using camphor as a PE. Therefore, increasing enhancement effects of terpenes are much more likely to be observed for hydrophilic or amphiphilic drugs rather than hydrophobic drugs. 

### 4.2. Lipophilicity of the Terpene

In addition to the lipophilicity of the drug, the lipophilicity of the terpene also plays an important role in determining the penetration enhancement effect. It is anticipated that hydrocarbon terpenes, such as limonene, exhibit a better penetration enhancement effect for lipophilic drug molecules, and conversely, the polar group containing terpenes, such as menthol, 1,8-cineole, provide a better penetration enhancement effect for hydrophilic drug molecules [11,28,42].

For highly lipophilic drugs, lipophilic terpenes with larger logP values seemed to be more effective as it was easier for them to mix with the SC intercellular lipids, thus, fluidizing or perturbing the integrity of the barrier function of the SC and, thereby, facilitating the skin penetration of the drugs. Among tested terpenes, anethole (log P = 3.39) was proved to be the most satisfactory PE for the permeation of lipophilic valsartan (log P = 4.5) followed by menthone (log P = 2.63). Eugenol (log P = 2.30) was the least effective terpene enhancer [17]. However, it should be noted that high lipophilicity of terpenes may have resulted in the decreased partitioning of ondansetron (log P = 2.07) into the SC [27].

For most drugs, amphiphilic terpenes such as nerolidol possess a high penetration enhancement effect because the amphiphilic structure is appropriate for the disruption of the highly organized lipid packing in the SC [16,30].

Extracting SC lipids is one of the key mechanisms of terpenes as PEs. The lipid extraction ability is enhanced for terpenes with high log P values (e.g., nerolidol) or terpenes that can form highly hydrophobic micellar structures with membrane lipids (e.g., limonene) [43].

### 4.3. Concentration of the Terpene

As presented in Table 2, the applied concentrations of the terpenes were in the range of 0.4%~15%. Most terpenes were applied in the range of 1%~5% in TDDS. For different terpenes, the optimum concentration may be different.

The penetration enhancement effect initially increased drastically with the increase in terpene concentration. However, the drug penetration was not significantly enhanced with the further increase in terpene concentration. The increase in ER values of the drug with the increase of terpene concentration is normally attributed to the ability of the terpene to modify the skin barrier properties, while the reduction of drug permeation at higher terpene concentrations could be attributed to the interaction between terpene and the drug [16].

### 4.4. Chemical Structure of the Terpene

Generally, the percutaneous absorption of hydrophilic drugs is better improved by terpenes with polar functional groups, which enable them to interact with the amide groups of the SC ceramides more competitively than do the terpenes with a carbonyl group. This leads to the disruption of the barrier provided by hydrogen bonding between lipid bilayers, and facilitate the diffusion of drugs through the SC [28].

A chain structure of a terpene may help increase the penetration enhancement effect better than a ring structure. It was found that terpenes with a ring structure, such as menthol and camphor, have less of an effect when compared to terpenes with a long chain alkyl structure, such as nerol and oleic acid [5]. Moreover, the chain molecule farnesol showed greater penetration enhancement effects for hydrophilic drugs than cyclic terpenes, probably due to its lower vaporization energy [16]. Terpenes with a low boiling point have relatively weaker intermolecular cohesive forces, which means the oxygen of the functional group is mostly free. Therefore, competitive hydrogen bonding between the functional groups of terpenes and the skin ceramides is facilitated [16].

The boiling point of a terpene is found to be inversely related to its skin penetration enhancement effects, as the penetration enhancement of zidovudine, 1,8-cineole with a boiling point of 173 °C was proved to be the most effective compared to other terpenes with higher boiling points (carvone: 230 °C; pulegone: 224 °C; menthone 210 °C; α-terpineol 217 °C; and menthol 215 °C) [44].

## 5. Skin Irritancy and Toxicity

Despite most PEs performing fairly well in TDDS, only a few of them have been approved for clinical application due to their skin toxicity or irritation. Generally, the potency of PEs parallels their potential for skin irritation and skin toxicity. It is challenging to maintain the balance between safety and potency of PEs.

Terpenes obtained from natural sources are generally considered to be less toxic compared to synthetic PEs, such as azone. The toxicities of terpenes were examined using an MTT assay in two skin cell lines including keratinocytes and fibroblasts. It was found that the IC_50_ values of borneol were markedly higher in both HaCaT keratinocytes (4.1150 ± 0.1489 mmol/L) and CCC-HSF-1 fibroblasts (4.9427 ± 0.2992 mmol/L) in comparison to those of the known standard enhancer azone (0.1169 ± 0.0086 mmol/L in HaCaT cells and 0.2425 ± 0.0233 mmol/L in CCC-HSF-1 cells), indicating that borneol had relatively low toxicity in skin cells [20]. Camphor, another monoterpene, was also proved to have low irritancy potential. In a cytotoxicity assay, the IC_50_ values of camphor were significantly higher in both keratinocytes (5.35 vs. 0.20 mmol/L) and fibroblasts (5.21 vs. 0.33 mmol/L) compared to azone [21]. Similar results were also obtained with limonene, terpinen-4-ol, and 1,8-cineole [25]. The IC_50_ values of limonene, terpinen-4-ol, and 1,8-cineole against HaCaT keratinocytes were determined to be 2.207 ± 0.035, 0.908 ± 0.033, and 1.400 ± 0.139 mg/mL, respectively. The IC_50_ values of limonene, terpinen-4-ol, and 1,8-cineole against CCC-ESF-1 fibroblasts were determined to be 0.938 ± 0.059, 0.745 ± 0.063, and 1.391 ± 0.113 mg/mL, respectively. Their previous studies [45] showed that the IC_50_ values of azone were 0.047 and 0.048 mg/mL in HaCaT cells and CCC-ESF-1 cells, respectively. In summary, the natural terpenes possessed relatively low skin irritation potential compared with azone. The effect of sesquiterpene nerolidol and various monoterpenes (α-terpineol, carvone, limonene, menthone, menthol, pulegone, and 1,8-cineole) on membrane fluidity in erythrocyte and fibroflast cells was studied and compared [46]. It was found that the effect of sesquiterpene was significantly greater than that of the monoterpenes. In both tests, nerolidol was among the most aggressive of terpenes and 1,8-cineole was among the least aggressive. The toxicity of sesquiterpenes seemed to be higher than that of monoterpenes.

Six terpene compounds, namely menthol, limonene, 1,8-cineole, methone, terpinen-4-ol, and pulegone, were proved to possess low cytoxicity in comparison with azone. Furthermore, the potential mechanisms of these terpenes were also investigated. Terpene penetration enhancers perhaps changed the membrane fluidity and potentials of HaCaT cells by altering the Ca^2+^ balance of the cell inside and outside, resulting in an increase in the drug transdermal absorption [47]. Determination of transepidermal water loss (TEWL) can be an effective index to represent the health of the skin barrier function. Consequently, TEWL was determined in the investigation to evaluate the skin irritation. The actual alteration in the TEWL (ΔTEWL) value was increased up to 10.32-fold and 24.05-fold after topical application of 5% borneol and azone, respectively. However, no significant differences were observed between 1% and 3% borneol and the control, suggesting the borneol had a relative weak impact on TEWL in a certain concentration range. The results indicated that borneol at an appropriate concentration did not produce obvious skin irritation [20]. However, although the non-oxidized terpenes were non-irritating, both linalool and limonene were found to be more irritating after oxidation compared with the pure terpene compounds [48]. Skin irritancy can be defined as reactions to a particular irritant that results in inflammation of the skin and itchiness. It should be noted that some terpenes, for example, α-bisabolol, can be applied as useful therapeutic candidates for the treatment of skin inflammation [49]. 

## 6. Discussion and Conclusions

Terpenes belong to a large class of the most abundant natural compounds and are commonly present in plants as constituents of essential oils. While it is difficult to synthesize novel chemical PEs, terpenes seem to possess great potential for use as PEs. Until now, at least 28 terpenes have been evaluated and applied as PEs in TDDS (Table 1). Among them, the most commonly used terpenes are 1,8-cineole, menthol, limonene, menthone, and nerolidol.

Compared to conventional synthetic PEs, natural terpenes have been shown to possess higher enhancement activity of both lipophilic and hydrophilic compounds. Results of in vitro skin permeation studies, in vivo pharmacokinetics, and pharmacological evaluation have demonstrated that terpenes can act as potential PEs due to their high enhancement activity and low toxicity. 

Interaction with SC intercellular lipids is the key factor determining the effectiveness of terpenes as PEs. The effect of terpenes on SC lipids mainly involves the interactions at two sites, namely the lipophilic tails of the intercellular lipids and the polar head groups. They can fluidize and/or extract the SC lipids to weaken the skin permeability barrier provided by the SC lipids. In the SC, a large number of ceramides are tightly arranged in the lipid bilayer due to the hydrogen bonding network. The tight hydrogen bonding network can be loosened by the terpenes with a functional group that can donate or accept a hydrogen bond. Consequently, most (89.29%) terpenes which can be used as PEs are oxygen-containing terpenes. Oxygen-containing and hydrocarbon terpenes could form complexes with drug molecules, which help in the SC partition of the drug. Their effect on the SC partition may depend on the lipophilicity of the drug. Furthermore, the physiological activity of terpenes in the living skin can also affect their efficacy as PEs.

The key factors affecting the enhancement effect are the lipophilicity of both terpenes and drug molecules. For most drugs, amphiphilic terpenes exert a high penetration enhancement effect because the amphiphilic structure is appropriate for the disruption of the highly organized lipid packing in the SC. Chain structure and low boiling point may help to improve the penetration enhancement effect of terpenes. Moreover, terpenes should be applied in optimum concentration. Most terpenes were applied in the concentration range of 1%~5% in TDDS. 

Revealed by skin cell viability assay and TEWL measurement, terpenes from natural sources are generally proven to be safer as PEs with very low irritancy potential compared to azone, the classic chemical skin PE. Until now, no correlation between skin toxicity and penetration enhancement effect has been found.

## Figures and Tables

**Table 1:** Terpenes applied as penetration enhancers (PEs).

Terpene	Type	Chemical Formula	MW ^a^	Log P	Boiling Point (°C)	Chemical Structure	Ref.
Anethole	Monoterpene	C_10_H_12_O	148.202	3.17	237.5	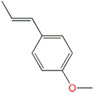	[16,17,18]
α-Bisabolol	Sesquiterpene	C_15_H_26_O	222.366	5.07	314.5	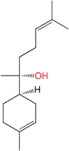	[19]
Borneol	Monoterpene	C_10_H_18_O	154.249	2.71	212	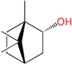	[16,19,20]
Camphor	Monoterpene	C_10_H_16_O	152.233	2.13	207.4	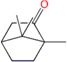	[19,21]
Carvacrol	Monoterpene	C_10_H_14_O	150.22	3.28	237.7	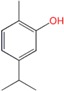	[22]
Carvone	Monoterpene	C_10_H_14_O	150.218	2.265	230.5	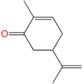	[23,24]
1,8-Cineole	Monoterpene	C_10_H_18_O	154.249	2.82	174	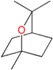	[11,15,22,23,24,25,26,27,28,29]
1,4-Cineole	Monoterpene	C_10_H_18_O	154.249	2.58	173.5	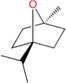	[16,28,29]
Cymene	Monoterpene	C_10_H_14_	134.22	4.02	173.9	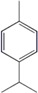	[24]
Eugenol	Monoterpene	C_10_H_12_O_2_	164.201	2.2	255	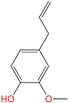	[17]
Farnesol	Sesquiterpene	C_15_H_26_O	222.366	5.31	283.4	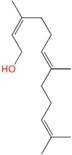	[16]
Fenchone	Monoterpene	C_10_H_16_O	152.23	2.13	193.5	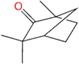	[24]
Geraniol	Monoterpene	C_10_H_18_O	154.249	3.28	229.5	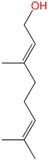	[16,24]
Limonene	Monoterpenes	C_10_H16	136.234	4.45	175.4	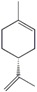	[11,16,23,24,25,27,30]
Linalool	Monoterpene	C_10_H_18_O	154.25	3.28	198.5	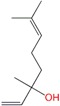	[26]
Menthol	Monoterpene	C_10_H_20_O	156.265	3.2	215.4	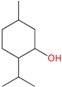	[11,16,22,23,26,31,32]
Menthone	Monoterpene	C_10_H_18_O	154.249	2.63	205	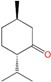	[17,22,26,31,32]
Nerolidol	Sesquiterpene	C_15_H_26_O	222.366	5.32	276	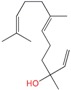	[16,24,26,27,32]
α-Pinene oxide	Monoterpene	C_10_H_16_O	152.23	2.11	188.6	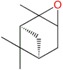	[26]
Pulegone	Monoterpene	C_10_H_16_O	152.233	2.56	224	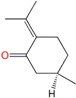	[32]
Rose oxide	Monoterpene	C_10_H_18_O	154.249	3.13	196.7	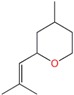	[28,29]
Safranal	Monoterpenes	C_10_H_14_O	150.218	2.9	217.3	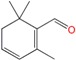	[28,29]
Terpinen-4-ol (4-terpinenol)	Monoterpenes	C_10_H_18_O	154.249	2.99	209	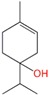	[24,25]
α-Terpineol	Monoterpenes	C_10_H_18_O	154.25	2.79	217.5	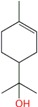	[24]
Tetra-hydrogeraniol	Monoterpene	C_10_H_22_O	158.281	3.64	212.5	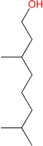	[16]
Thymol	Monoterpenes	C_10_H_14_O	150.22	3.28	233	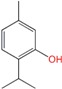	[21,23]
Valen-cene	Sesquiterpene	C_15_H_24_	204.351	6.28	270.5	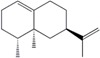	[27,28]
Verbenon-e	Monoterpenes	C_10_H_14_O	150.22	1.97	227.5	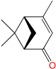	[23]

Note: ^a^ MW = molecular weight.

**Table 2:** Skin penetration enhancement effect of terpenes applied as PEs.

Terpene	Drug	Parameters	Vehicle (Terpene Concentration)	Skin	Receptor Liquid	ER ^a^	Proposed Mechanism	Ref.
Anethole	Propranolol hydrochloride	log P = 1.53, MW = 295.804	hydroxypropyl methylcellulose (HPMC) gel (3%)	Piglet abdominal skin	pH 7.4 PBS	1.8	Enhancing diffusion through the intercellular lipids	[16]
	Valsartan	log P = 4.5, MW = 435.519	Ethanol:pH 7.4 isotonic PBS = 40:60 (1%)	Rat abdominal skin	Ethanol:pH 7.4 isotonic PBS = 40:60	4.4	Extracting stratum corneum (SC) lipids and breaking the hydrogen bonds	[17]
	Etodolac	log P = 3.59, MW = 287.35	Carboxyl methyl cellulose (CMC)-Na gel (1%)	Rat abdominal skin	pH 7.4 PBS	1.52	Interacting with lipid components of the SC	[18]
α-Bisabolol	Propranolol hydrochloride	log P = 1.53, MW = 295.804	66.7% ethanol (5%)	Rat dorsal skin	pH 7.4 PBS	6.29	Increasing lipid fluidity and improving the partition into the SC	[19]
Borneol	5-Fluorouracil	log P = −0.95, MW = 130.08	PG:water = 70:30 (1%, 3%, 5%)	Rat abdominal skin	1% Brij98 in isotonic PBS (pH 7.2)	1% = 3.77 3% = 7.49 5% = 10.57	Disrupting and extracting part of SC intercellular lipids	[20]
	Antipyrine	log P = 0.23, MW = 188.23				1% = 6.51 3% = 19.18 5% = 32.84		
	Aspirin	log P = 1.23, MW = 180.04				1% = 5.12 3% = 13.31 5% = 19.81		
	Salicyclic acid	log P = 2.25, MW = 138.12				1% = 2.40 3% = 8.10 5% = 12.76		
	Ibuprofen	log P = 3.51, MW = 206.28				1% = 1.55 3% = 5.18 5% = 9.78		
	Propranolol hydrochloride	log P = 1.53, MW = 295.804	HPMC gel (3%)	Piglet abdominal skin	pH 7.4 PBS	1.5	Enhancing diffusion through the intercellular lipids	[16]
	Propranolol hydrochloride	log P = 1.53, MW = 295.804	66.7% ethanol (5%)	Rat dorsal skin	pH 7.4 PBS	5.01	Increasing lipid fluidity and improving the partition into the SC	[19]
Camphor	Indometacin	log P = 3.80, MW = 357.79	PG:water = 70:30 (3%)	Rat abdominal skin	Isotonic pH 7.2 PBS	3.97	Perturbing the regular organization of SC lipids or directly extracting part of the SC lipids; improving the partition into the SC	[21]
	Lidocaine	log P = 2.56, MW = 234.34				5.68		
	Aspirin	log P = 1.23, MW = 180.04				9.82		
	Antipyrine	log P = 0.23, MW = 188.23				17.80		
	Tegafur	log P = −0.48, MW = 200.17				15.98		
	5-Fluorouracil	log P = −0.95, MW = 130.08				11.87		
	Propranolol hydrochloride	log P = 1.53, MW = 295.804	Ethanol:water = 2:1 (5%)	Rat dorsal skin	pH 7.4 PBS	3.67	Increasing lipid fluidity and improving the partition into the SC	[19]
Carvacrol	Corticosterone	log P = 1.76, MW = 346.46	pH 7.4 PBS (1.2/1.8/3.0/3.5 mM)	Human epidermal membrane	pH 7.4 PBS	3.9 (1.2 mM) 6.6 (1.8 mM) 9.5 (3.0 mM) 14.7 (3.5 mM)	SC intercellular lipid fluidization	[22]
Carvone	Genistein	log P = 2.94, MW = 270.237	MC gel (0.4%)	Human skin	0.01 MPBS (pH 7.4):ethanol = 8:2	4.78	Disrupting the SC lipid bilayers	[23]
	Hydrocortisone	log P = 1.43, MW = 362.46	HPMC gel (2%)	Hairless mouse abdominal skin	Isotonic PBS (pH 7.2)	13.1	Disrupting the hydrophobic lipid packing of the SC	[24]
1,8-Cineole (eucalyptol)	Corticosterone	log P = 1.76, MW = 346.46	pH 7.4 PBS (2.0/3.0/4.0 mM)	Human epidermal membrane	pH 7.4 PBS	1.9 (2.0 mM) 3.2 (3.0 mM) 3.6 (4.0 mM)	SC intercellular lipid fluidization	[22]
	Genistein	log P = 2.94, MW = 270.237	MC gel (0.4%)	Human skin	0.01 MPBS (pH 7.4):ethanol = 8:2	7.41	Disrupting the SC lipid bilayers	[23]
	Hydrocortisone	log P = 1.43, MW = 362.46	HPMC gel (2%)	Hairless mouse abdominal skin	Isotonic PBS (pH 7.2)	14.5	Disrupting the hydrophobic lipid packing of the SC	[24]
	Osthole	log P = 3.85, MW = 244.34	PG:water = 80:20 (3%)	Rat abdominal skin	pH 7.2 PBS, 3% Brij98 added for osthole	2.27	Perturbing and extracting the SC lipids; altering the keratin conformation to some extent	[25]
	Tetramethylpyr-azine	log P = 2.34, MW = 136.20				2.16		
	Ferulic acid	log P = 1.26, MW = 194.18				1.22		
	Puerarin	log P = −0.35, MW = 432.38				0.60		
	Geniposide	log P = −1.01, MW = 388.37				2.80		
	Lomerizine dihydro-chloride	log P = 4.8, MW = 541.457	PG (10%)	Hairless mouse dorsal skin	0.5% Tween80 in 0.9% NaCl solution	Flux = 28.8 ± 8.5 µg/cm^2^/h control = 0	Increasing the fluidity of SC lipids; causing disorder of the stacking arrangement of the lipid bilayers	[26]
	Ondansetron hydrochloride	log P = 2.07, MW = 329.824	Chitosan gel film (1%)	Porcine dorsal skin	0.9% Saline solution	3.23	Increasing lipid fluidity of the SC	[27]
	Valsartan	log P = 4.5, MW = 435.519	Ethanol:isotonic IPB (pH 7.4) = 40:60 (0.5%, 0.75%, 1%, 3%, 5%)	Rat abdominal skin	Ethanol:isotonic IPB (pH 7.4) = 40:60	2.15 (0.5%) 2.34 (0.75%) 6.4 (1%) 4.2 (3%) 3.7 (5%)	Extraction of SC lipids and keratin denaturation in the SC	[28]
	Valsartan	log P = 4.5, MW = 435.519	Carbopol940 gel (1%)	Rat abdominal skin	ethanol:isotonic PBS (pH 7.4) = 40:60	4.53	Disrupting the intercellular packing of the SC lipids	[15]
	Propranolol hydrochloride	log P = 1.53, MW = 295.804	Ethanol:isotonic PBS (pH 7.4) = 20:80 (0.5%, 0.75%, 1%)	Rat abdominal skin	PBS pH 7.4	1.11 (0.5%) 1.20 (0.75%) 2.07 (1%)	Extraction and disruption of SC lipid bilayers and keratin denaturation in the SC	[29]
	Bufalin	log P = 2.78, MW = 386.5	PG/water = 50/50 (5%)	Hairless mouse dorsal skin	PEG:PG:water = 40:30:30	17.1	Modifying the intercellular packing and disrupting highly ordered structure of lipids	[13]
1,4-Cineole	Propranolol hydrochloride	log P = 1.53, MW = 295.804	HPMC gel (3%)	Piglet abdominal skin	pH 7.4 PBS	2.7	Enhancing diffusion through the intercellular lipids	[16]
	Propranolol hydrochloride	log P = 1.53, MW = 295.804	Ethanol:isotonic PBS (pH 7.4) = 20:80 (0.5%, 0.75%, 1%)	Rat abdominal skin	PBS pH 7.4	1.38 (0.5%) 1.95 (0.75%) 3.07 (1%)	Extraction and disruption of SC lipid bilayers and keratin denaturation in the SC	[29]
	Valsartan	log P = 4.5, MW = 435.519	Ethanol:isotonic PBS (pH 7.4) = 40:60 (0.5%, 0.75%, 1%, 3%, 5%)	Rat abdominal skin	Ethanol:isotonic PBS (pH 7.4) = 40:60	2.77 (0.5%) 3.39 (0.75%) 7.4 (1%) 6.1 (3%) 5.4 (5%)	Extraction of SC lipids and keratin denaturation in the SC	[28]
Cymene	Hydrocortisone	log P = 1.43, MW = 362.46	HPMC gel (2%)	Hairless mouse abdominal skin	Isotonic PBS (pH 7.2)	22.9	Disrupting the hydrophobic lipid packing of the SC	[24]
Eugenol	Valsartan	log P = 4.5, MW = 435.519	Ethanol:pH 7.4 isotonic PBS = 40:60 (1%)	Rat abdominal skin	Ethanol:pH 7.4 isotonic PBS = 40:60	3.0	Extracting SC lipids and breaking the hydrogen bonds	[17]
Farnesol	Propranolol hydrochloride	log P = 1.53, MW = 295.804	HPMC gel (3%)	Piglet abdominal skin	pH 7.4 PBS	3.9	Enhancing diffusion through the intercellular lipids	[16]
Fenchone	Hydrocortisone	log P = 1.43, MW = 362.46	HPMC gel (2%)	Hairless mouse abdominal skin	Isotonic PBS (pH 7.2)	10.1	Disrupting the hydrophobic lipid packing of the SC	[24]
Geraniol	Propranolol hydrochloride	log P = 1.53, MW = 295.804	HPMC gel (3%)	Piglet abdominal skin	pH 7.4 PBS	2.8	Enhancing diffusion through the intercellular lipids	[16]
	Hydrocortisone	log P = 1.43, MW = 362.46	HPMC gel (2%)	Hairless mouse abdominal skin	Isotonic PBS (pH 7.2)	16.9	Disrupting the hydrophobic lipid packing of the SC	[24]
Limonene	Terbinafine	log P = 3.3, MW = 291.43	Carbopol 934P gel (5%)	Porcine dorsal skin	pH 5.8 PBS	1.36	Lipid bilayer disruption in the SC	[30]
	Bufalin	log P = 2.78, MW = 386.5	PG/water = 50/50 (5%)	Hairless mouse dorsal skin	PEG/PG/water = 40/30/30	22.2	Increasing the skin diffusivity by modifying the intercellular packing and disrupting highly ordered structure of lipids	[11]
	Hydrocortisone	log P = 1.43, MW = 362.46	HPMC gel (2%)	Hairless mouse abdominal skin	Isotonic PBS (pH 7.2)	28.4	Disrupting the hydrophobic lipid packing of the SC	[24]
	Osthole	log P = 3.85, MW = 244.34	PG:water = 80:20 (3%)	Rat abdominal skin	pH 7.2 PBS, 3% Brij98 added for osthole	10.55	Perturbing and extracting the SC lipids	[25]
	Tetramethylpyrazine	log P = 2.34, MW = 136.20				9.61		
	Ferulic acid	log P = 1.26, MW = 194.18				53.78		
	Puerarin	log P = −0.35, MW = 432.38				18.40		
	Geniposide	log P = −1.01, MW = 388.37				5.70		
	Ondansetron hydrochloride	log P = 2.07, MW = 329.824	Chitosan gel film (1%)	Porcine dorsal skin	0.9% Saline solution	0.94	-	[27]
	Propranolol hydrochloride	log P = 1.53, MW = 295.804	HPMC gel (3%)	Piglet abdominal skin	pH 7.4 PBS	2.6	Enhancing diffusion through the intercellular lipids	[16]
	Genistein	log P = 2.94, MW = 270.237	Methyl cellulose (MC) gel (0.4%)	Human skin	0.01 MPBS (pH 7.4):ethanol = 8:2	1.73	Disrupting the lipid bilayers of the SC	[23]
Linalool	Lomerizine dihydrochloride	log P = 4.8, MW = 541.457	PG (10%)	Hairless mouse dorsal skin	0.5% Tween80 in 0.9% NaCl solution	Flux = 16.6 ± 4.1 µg/cm^2^/h control = 0	Increasing the fluidity of SC lipids; causing disorder of the stacking arrangement of the lipid bilayers	[26]
Menthol	Ligustrazine hydrochloride	log P = 1.26, MW = 172.655	Film composed of PVA and CMC-Na (3%)	Porcine dorsal skin	Water	Flux = 6.30 µg/cm^2^/h Azone = 0.74 µg/cm^2^/h	Disturbing and extracting SC lipids and hydrogen bond connection	[31]
	Osthole	log P = 3.85, MW = 244.34	PG:water = 80:20 (3%)	Rat abdominal skin	Isotonic 0.01 M PBS (pH 7.2)	1.21	Disordering the ordered organization of SC lipids and extracting part of the SC lipids	[32]
	Tetramethylpyrazine	log P = 2.34, MW = 136.20				3.92		
	Ferulic acid	log P = 1.26, MW = 194.18				35.32		
	Puerarin	log P = −0.35, MW = 432.38				66.40		
	Geniposide	log P = −1.01, MW = 388.37				32.20		
	Genistein	log P = 2.94, MW = 270.237	MC gel (0.4%)	Human skin	0.01 MPBS (pH 7.4):ethanol = 8:2	9.59	Disrupting the lipid bilayers of the SC	[23]
	Corticosterone	log P = 1.76, MW = 346.46	pH 7.4 PBS (1.0/1.5/2.0 mM)	Human epidermal membrane	pH 7.4 PBS	2.8 (1.0 mM) 3.8 (1.5 mM) 4.9 (2.0 mM)	SC intercellular lipid fluidization	[22]
	Lomerizine dihydrochloride	log P = 4.8, MW = 541.457	PG (10%)	Hairless mouse dorsal skin	0.5% Tween80 in 0.9% NaCl solution	Flux = 28.4 ± 6.6 µg/cm^2^/h control = 0	Increasing the fluidity of SC lipids; causing disorder of the stacking arrangement of the lipid bilayers	[26]
	Propranolol hydrochloride	log P = 1.53, MW = 295.804	HPMC gel (3%)	Piglet abdominal skin	pH 7.4 PBS	3.7	Enhancing diffusion through the intercellular lipids	[16]
	Bufalin	log P = 2.78, MW = 386.5	PG/water = 50/50 (5%)	Hairless mouse dorsal skin	PEG/PG/water = 40/30/30	15.3	Increasing the skin diffusivity by modifying the intercellular packing and disrupting highly ordered structure of lipids	[11]
Menthone	Valsartan	log P = 4.5, MW = 435.519	Ethanol:pH 7.4 isotonic PBS = 40:60 (1%)	Rat abdominal skin	Ethanol:pH 7.4 isotonic PBS = 40:60	4.0	Extracting SC lipids and breaking the hydrogen bonds	[17]
	Osthole	log P = 3.85, MW = 244.34	PG:water = 80:20 (3%)	Rat abdominal skin	Isotonic 0.01 M PBS (pH 7.2)	5.82	Disordering the ordered organization of SC lipids and extracting part of the SC lipids	[32]
	Tetramethylpyrazine	log P = 2.34, MW = 136.20				8.54		
	Ferulic acid	log P = 1.26, MW = 194.18				20.42		
	Puerarin	log P = −0.35, MW = 432.38				293.80		
	Geniposide	log P = −1.01, MW = 388.37				31.60		
	Lomerizine dihydro-chloride	log P = 4.8, MW = 541.457	PG (10%)	Hairless mouse dorsal skin	0.5% Tween80 in 0.9% NaCl solution	Flux = 20.6 ± 2.5 µg/cm^2^/h control = 0	Increasing the fluidity of SC lipids; causing disorder of the stacking arrangement of the lipid bilayers	[26]
	Corticosterone	log P = 1.76, MW = 346.46	pH7.4 PBS (2.0/2.6/3.0 mM)	Human epidermal membrane	pH 7.4 PBS	3.8 (2.0 mM)	SC intercellular lipid fluidization	[22]
						4.6 (2.6 mM)		
						5.9 (3.0 mM)		
	Ligustrazine hydrochloride	log P = 1.26, MW = 172.655	Film composed of PVA and CMC-Na (3%)	Porcine dorsal skin	Water	Flux = 5.37 µg/cm^2^/h Azone = 0.74 µg/cm^2^/h	Disturbing and extracting SC lipids and hydrogen bond connection	[31]
Nerolidol	Terbinafine	log P = 3.3, MW = 291.43	Carbopol 934P gel (5%)	Porcine dorsal skin	pH 5.8 PBS	4.13	Lipid bilayer disruption in the SC	[30]
	Lomerizine dihydrochloride	log P = 4.8, MW = 541.457	PG (10%)	Hairless mouse dorsal skin	0.5% Tween80 in 0.9% NaCl solution	Flux = 14.2 ± 3.0 µg/cm^2^/h control = 0	Increasing the fluidity of SC lipids; causing disorder of the stacking arrangement of the lipid bilayers	[26]
	Ondansetron hydrochloride	log P = 2.07, MW = 329.24	Chitosan gel film (1%)	Porcine dorsal skin	0.9% Saline solution	0.85	-	[27]
	Propranolol hydrochloride	log P = 3.77, MW = 295.804	HPMC gel (3%)	Piglet abdominal skin	pH 7.4 PBS	3.4	Enhancing diffusion through the intercellular lipids	[16]
	Hydrocortisone	log P = 1.43, MW = 362.46	HPMC gel (2%)	Hairless mouse abdominal skin	Isotonic PBS (pH 7.2)	35.3	Disrupting the hydrophobic lipid packing of the SC	[24]
α-Pinene oxide	Lomerizine dihydro-chloride	log P = 4.8, MW = 541.457	PG (10%)	Hairless mouse dorsal skin	0.5% Tween80 in 0.9% NaCl solution	Flux = 23.1 ± 1.9 µg/cm^2^/h control = 0	Increasing the fluidity of SC lipids; causing disorder of the stacking arrangement of the lipid bilayers	[26]
Pulegone	Osthole	log P = 3.85, MW = 244.34	PG:water = 80:20 (3%)	Rat abdominal skin	Isotonic 0.01 M PBS (pH 7.2)	2.87	Extracting part of the SC lipids	[32]
	Tetra-methylpyrazine	log P = 2.34, MW = 136.20				2.67		
	Ferulic acid	log P = 1.26, MW = 194.18				3.07		
	Puerarin	log P = −0.35, MW = 432.38				2.60		
	Geniposide	log P = −1.01, MW = 388.37				2.70		
Rose oxide	Valsartan	log P = 4.5, MW = 435.519	Ethanol:isotonic PBS (pH 7.4) = 40:60 (0.5%, 0.75%, 1%, 3%, 5%)	Rat abdominal skin	Ethanol:isotonic PBS (pH 7.4) = 40:60	1.78 (0.5%) 2.11 (0.75%) 5.7 (1%) 6.1 (3%) 6.4 (5%)	Extraction of SC lipids and keratin denaturation in the SC	[28]
	Propranolol hydrochloride	log P = 3.77, MW = 295.804	Ethanol:isotonic PBS (pH 7.4) = 20:80 (0.5%, 0.75%, 1%)	Rat abdominal skin	PBS pH 7.4	1.06 (0.5%) 1.13 (0.75%) 1.71 (1%)	Extraction and disruption of SC lipid bilayers and keratin denaturation in the SC	[29]
Safranal	Valsartan	log P = 4.5, MW = 435.519	Ethanol:isotonic PBS (pH 7.4) = 40:60 (0.5%, 0.75%, 1%, 3%, 5%)	Rat abdominal skin	Ethanol:isotonic PBS (pH 7.4) = 40:60	1.49 (0.5%) 2.05 (0.75%) 3.7 (1%) 3.4 (3%) 3.0 (5%)	Extraction of SC lipids and keratin denaturation in the SC	[28]
	Propranolol hydrochloride	log P = 3.77, MW = 295.804	Ethanol:isotonic PBS (pH7.4) = 20:80 (0.5%, 0.75%, 1%)	Rat abdominal skin	PBS pH 7.4	1.03 (0.5%) 1.08 (0.75%) 1.20 (1%)	Extraction and disruption of SC lipid bilayers and keratin denaturation in the SC	[29]
Terpinen-4-ol (4-terpinenol)	Osthole	log P = 3.85, MW = 244.34	PG:water = 80:20 (3%)	Rat abdominal skin	pH 7.2 PBS, 3% Brij98 added for osthole	1.90	Perturbing and extracting the SC lipids	[25]
	Tetramethylpyrazine	log P = 2.34, MW = 136.20				1.64		
	Ferulic acid	log P = 1.26, MW = 194.18				2.02		
	Puerarin	log P = −0.35, MW = 432.38				0.40		
	Geniposide	log P = −1.01, MW = 388.37				2.00		
	Hydrocortisone	log P = 1.43, MW = 362.46	HPMC gel (2%)	Hairless mouse abdominal skin	Isotonic PBS (pH 7.2)	11.3	Disrupting the hydrophobic lipid packing of the SC	[24]
α-Terpineol	Hydrocortisone	log P = 1.43, MW = 362.46	HPMC gel (2%)	Hairless mouse abdominal skin	Isotonic PBS (pH 7.2)	13.3	Disrupting the hydrophobic lipid packing of the SC	[24]
Tetra-hydrogeraniol	Propranolol hydrochloride	log P = 3.77, MW = 295.804	HPMC gel (3%)	Piglet abdominal skin	pH 7.4 PBS	3.3	Enhancing diffusion through the intercellular lipids	[16]
Thymol	Hydrocortisone	log P = 1.43, MW = 362.46	HPMC gel (2%)	Hairless mouse abdominal skin	Isotonic PBS (pH 7.2)	11.0	Disrupting the hydrophobic lipid packing of the SC	[24]
	Corticosterone	log P = 1.76, MW = 346.46	pH 7.4 PBS (1.0/1.8/3.0/4.0 mM)	Human epidermal membrane	pH 7.4 PBS	3.1 (1.0 mM) 5.5 (1.8 mM) 10.9 (3.0 mM) 17.2 (4.0 mM)	SC intercellular lipid fluidization	[22]
Valencene	Valsartan	log P = 4.5, MW = 435.519	Ethanol:isotonic PBS (pH 7.4) = 40:60 (0.5%, 0.75%, 1%, 3%, 5%)	Rat abdominal skin	Ethanol:isotonic PBS (pH 7.4) = 40:60	1.96 (0.5%) 2.14 (0.75%) 6.2 (1%) 4.3 (3%) 2.6 (5%)	Extraction of SC lipids and keratin denaturation in the SC	[28]
	Propranolol hydrochloride	log P = 3.77, MW = 295.804	Ethanol:isotonic PBS (pH 7.4) = 20:80 (0.5%, 0.75%, 1%)	Rat abdominal skin	PBS pH 7.4	1.19 (0.5%) 1.26 (0.75%) 2.20 (1%)	Extraction and disruption of the SC lipid bilayers and keratin denaturation in the SC	[29]
Verbenone	Hydrocortisone	log P = 1.43, MW = 362.46	HPMC gel (2%)	Hairless mouse abdominal skin	Isotonic PBS (pH 7.2)	11.5	Disrupting the hydrophobic lipid packing of the SC	[24]

Note: ^a^ The enhancement ratio (ER) was calculated as follows: ER = flux of the drug with terpene/ flux of the drug without terpene.
